# Dog-mediated active-leisure engagement and organised canine sport: a cross-sectional analysis of Hungarian dog owners

**DOI:** 10.3389/fvets.2026.1912638

**Published:** 2026-09-25

**Authors:** Anetta Müller, Attila Lengyel, Katalin Mező, Éva Bácsné Bába, Elena Bendíková, Réka Pálinkás, Ricardo Uvinha, Ferenc Mező

**Affiliations:** 1 Faculty of Economics and Business, Institute of Sports Economics and Management, University of Debrecen, Debrecen, Hungary; 2 Department of Physical Education and Sports, Faculty of Education, Katolicka univerzita v Ruzomberku, Ružomberok, Slovakia; 3 Department of Physical Education and Sports, Faculty of Education, Catholic University in Ružomberok, Ružomberok, Slovakia; 4 Faculty of Economics and Business, Institute of Sports Economics and Management, University of Debrecen, Debrecen, Hungary; 5 School of Arts, Sciences and Humanities, University of São Paulo, São Paulo, Brazil; 6 Faculty of Pedagogy, Institute of Psychology, Eszterházy Károly Catholic University, Eger, Hungary

**Keywords:** active leisure, canine sport, dog ownership, human animal bond, Hungary, logistic regression, physical activity

## Abstract

**Background:**

Companion dogs are widely seen as a route to physical activity, yet owners differ greatly in how actively they engage their animals, and average owner–non-owner comparisons obscure this variation. We asked how strongly an owner’s dog-mediated active-leisure engagement is associated with organised physical activity, and whether that engagement is patterned by place of residence and by the economic priority owners assign to their dog’s health.

**Methods:**

Cross-sectional survey data from 954 Hungarian adult dog owners were analysed. Engagement was measured as the mean of ten five-point items spanning affective, dispositional, and behavioural facets (Cronbach’s *α* = 0.84; McDonald’s *ω* = 0.84). Lifetime participation in organised canine sport was modelled in binary logistic regression with engagement (per standard deviation), gender, capital-city residence, and age as predictors; the breadth of participation, residence, and relative health-related expenditure were examined as further outcomes. Non-linearity of the engagement effect was tested, and a latent profile analysis is reported as a supplementary comparison.

**Results:**

Engagement was approximately normally distributed (*M* = 2.99, SD = 0.83) and essentially unidimensional. Owners who had taken part in canine sport scored 0.79 standard deviations higher on engagement than those who had not. In the adjusted model, each standard deviation of engagement was associated with 2.27 times the odds of lifetime canine sport participation [95% CI (1.94, 2.64)]; the predicted probability rose from 26% one standard deviation below the mean to 65% one standard deviation above it, with a plateau above roughly 3.5 on the 1–5 scale. Capital-city residence predicted participation on its own (OR = 1.89) but not once engagement was included [OR = 1.36, 95% CI (0.87, 2.10)]. Engagement was higher in the capital than elsewhere and was positively associated with prioritising the dog’s health in household spending. Among participants, the number of distinct sports undertaken was only weakly related to engagement.

**Discussion:**

Among owners, engagement rather than ownership distinguishes the physically active from the inactive, and the association is continuous across the full range of the scale rather than confined to a distinct subgroup. The potential for promoting active living through dogs therefore lies in shifting the large mid-range of the engagement distribution, where organised canine activity is still uncommon.

## 1 Introduction

Regular physical activity is one of the most dependable contributors to physical and mental health across adulthood, and the benefits accrue even at modest, sustained doses well below the level of formal exercise (1). For many people the obstacle is not recognising that activity matters but finding a reliable way to fit it into ordinary life, where competing demands tend to crowd out discretionary movement. Companion dogs offer one of the few everyday arrangements that build physical activity into routine almost by default: a dog needs walking, playing with, and time outdoors, and meeting those needs pulls the owner into activity as a matter of course (2). It is largely for this reason that dog ownership has held the sustained attention of researchers concerned with active living. The animal functions as a standing, daily prompt to move—one embedded in affection and obligation rather than in willpower or scheduling (2).

The connection between owning a dog and being active is, however, looser than the intuition suggests. Reviews of the dog-walking evidence report that a substantial minority of owners walk their dogs seldom or never, so that the average activity advantage of owners over non-owners conceals wide variation inside the owning population itself (2, 3). The gap between potential and realised activity is the analytically interesting part: a dog creates an opportunity for movement, but whether that opportunity is taken up varies enormously from owner to owner. Several studies suggest that the decisive factor is not ownership as a status but the quality of the owner–dog relationship—how strong the attachment is, how central the dog is to the person’s leisure, and what the pair actually do together (4). The strength of the human–animal bond, in particular, has been linked to more frequent shared activity and a greater willingness to organise daily life around the dog (4). Read against this evidence, treating owners as a single undifferentiated group means surrendering precisely the contrasts that matter most for active living.

Dog-mediated leisure also spans a strikingly broad range of intensity and structure. At one end sit informal, low-effort routines, such as a brief walk around the neighbourhood that asks little of either party. At the other lie organised and physically demanding pursuits—agility, canicross, flyball, mantrailing, obedience, and dog dance among them—that have grown into a structured leisure sector with clubs, training regimes, governing bodies, and competition calendars (5, 6). These canine sports deserve particular attention from an active-living standpoint. They involve sustained, often vigorous physical activity undertaken jointly by owner and dog; they are typically embedded in a social and organisational structure that supports adherence; and they demand a level of commitment that ordinary walking does not. Yet the literature on dogs and health has concentrated overwhelmingly on walking, with the result that organised canine sport remains comparatively unexamined as a form of physical activity in its own right (2, 3). Treating it as an outcome, rather than as a niche curiosity, is one way to extend what is known about how dogs contribute to active lifestyles.

How actively an owner engages a dog is unlikely to rest on any single cause. Dispositional factors plausibly matter—a general orientation toward active leisure, and an openness to taking up new activities (7, 8). So do affective factors, organised around the human–animal bond and the place the animal holds in the owner’s emotional life (4, 6). And so do structural factors, beginning with where the owner lives. Organised canine sport depends on clubs, training grounds, instructors, and events that are distributed unevenly across a country, tending to concentrate in larger urban centres; an owner’s access to that infrastructure is therefore partly a matter of geography rather than motivation (6). Engagement may also leave an economic trace, visible in how owners weigh spending on the dog’s health against spending on their own—a small but telling indicator of where the animal sits among a household’s priorities. These dispositional, affective, structural, and economic strands are usually studied one at a time. Bringing them together around a single, empirically grounded measure of engagement, and relating that measure directly to organised physical activity, is part of what the present study attempts.

The Hungarian setting makes this an informative case. Dog keeping is unusually common: representative surveys by the Animal Protection Legal, Analytical and Methodological Center at the University of Veterinary Medicine Budapest record dog-owning households rising from 36% in 2018 to more than half by 2021, and to 56% by 2023—among the highest rates in the European Union (9). Industry figures place the national pet-dog population in the region of 2.8 million (10). Ownership here is not only a matter of companionship but is woven into owners’ active leisure: in a Hungarian survey of 220 owners, 77.3% reported walking with their dog and roughly 26% exercising together with it (11). The activity has measurable stakes—joint owner–dog activity and participation in dog sport have been associated with lower odds of canine overweight in a large Hungarian sample (12)—and recent work has begun to map the costs and benefits owners attach to keeping a dog (13). The professional ecology around active dog keeping is also developing, if patchily: small-animal physiotherapy, for instance, was available in the practices of 28% of surveyed Hungarian veterinarians, with a further 24% planning to introduce it (14). Hungary thus combines a large, engaged owner base with a supporting infrastructure that is emerging but spread irregularly across the country—the conditions under which differences in active engagement, and in access to organised canine activity, are most likely to matter.

A methodological habit of the field is a further reason these strands tend to stay apart. Most research on dogs and physical activity has estimated average associations between ownership and activity, frequently resting on the blunt contrast between owners and non-owners (2, 3). That contrast answers a different question from the one we pose. We ask, within the owning population, how strongly the degree of an owner’s dog-mediated active-leisure engagement is associated with organised physical activity, and whether that engagement is itself patterned by structural and economic circumstances. Answering it requires a measure that captures engagement as a graded quantity across affective, dispositional, and behavioural facets, and an analysis that relates that quantity directly to activity outcomes. The value of typological approaches for active-living research has been demonstrated recently in the study of children’s leisure, where latent class models revealed distinct activity profiles with sharply different physical-activity content (15–18); whether adult, dog-mediated leisure engagement forms comparable discrete types or a single continuum is, however, an empirical question, and one the present study addresses directly.

Against this background, the present study examines how dog-mediated active-leisure engagement relates to organised physical activity among Hungarian dog owners. Engagement is measured with a ten-item index, and the focal outcome is participation and breadth of involvement in canine sport, deliberately measured separately from the engagement items so that the association is a genuine test of criterion validity rather than a restatement of the measure. We then ask whether engagement differs on two contextual dimensions that bear directly on active living: access, indexed by metropolitan residence, and the economic priority owners assign to their dog’s health relative to their own. The design is cross-sectional and the analysis associative throughout. We describe how engagement relates to physical activity at a single point in time, and we make no claim about whether dogs lead their owners to become more active.

## 2 Methods

### 2.1 Design and participants

We used a cross-sectional survey design. Adult dog owners living in Hungary completed a structured, self-administered online questionnaire in spring 2024. The design is descriptive and associative rather than explanatory: the goal was to measure the degree of dog-mediated active-leisure engagement among owners and then to ask how strongly it is associated with organised physical activity—specifically participation in canine sport—and with a set of structural and economic indicators measured at the same time. We make no claim about within-person change or causation. Every association reported below is a between-person difference observed at a single point in time.

Participants were recruited through an online panel using non-probability quota sampling, with quotas on gender, age group, and place of residence set to approximate the demographic profile of the Hungarian adult dog-owning population (19, 20). The recruitment target was 900 completed responses, in line with conventions for large-scale survey work in leisure research (21, 22). After excluding respondents who did not own a dog at the time of data collection, the analytic sample was *n* = 954. Completion took roughly fifteen minutes, and no incentive beyond access to the aggregate findings was offered.

### 2.2 Measures

Table 1 summarises the variables used in the analysis: the ten items that form the engagement index, the canine sport outcomes, and the structural and economic variables.

**Table 1:** Active-leisure engagement items and outcome/validation variables.

Set	Variables	Format	Role
Leisure-orientation items	Active-leisure preference; leisure time with dog; preference for animal company; canine-sport preference; daily sport; time in nature; openness to novelty	Likert 1–5	Engagement index items
Activity items	Frequency of running, hiking, trekking with the dog	Likert 1–5	Engagement index items
Canine sport (primary outcome)	Lifetime participation in 13 organised canine sports (per sport; binary any-participation; composite count 0–13)	Dichotomous; count	Outcome
Structural/sociodemographic	Place of residence; gender; age group	Categorical/ordinal	Covariate
Relative expenditure	Own vs. dog health expenditure (*n* = 533)	Categorical	Outcome

#### 2.2.1 Active-leisure engagement index

Ten items measured dog-mediated active-leisure engagement. The set was fixed in advance on two grounds. Theoretically, the items had to span the affective component of the human–animal bond, the dispositional component of an active-leisure orientation, and the behavioural component of dog-mediated physical activity (23–25). Distributionally, an item was retained only if it carried enough response variance to discriminate among owners, since a near-constant item contributes nothing to a measure of individual differences (26).

Seven items came from a leisure-orientation block scored from 1 (not *at all* characteristic) to 5 (fully characteristic): active-leisure preference, leisure time spent with the dog, preference for spending leisure time in the company of animals, preference for canine sport, daily sport engagement, time spent in nature, and openness to novelty. Three further items measured how often the owner ran, hiked, and trekked with the dog, each on a 1 (never) to 5 (several *times per* week) frequency scale. Three other dog-mediated activities—hunting, winter sports, and cycling—were left out under the variance criterion set in advance: 88% of respondents never hunted with their dog and 80% never took part in winter activities. The engagement index is the unweighted mean of the ten items on their original 1–5 metric; in regression models it is entered per standard deviation. For the facet-level analysis, three subscales were formed from the same items: affective (leisure time with the dog, preference for animal company), dispositional (active-leisure preference, canine-sport preference, daily sport, time in nature, openness to novelty), and behavioural (running, hiking, and trekking with the dog).

#### 2.2.2 Canine sport participation and breadth

Organised physical activity was captured through lifetime participation in thirteen canine sports (for example agility, flyball, obedience, mantrailing, canicross, and dog dance), each recorded as a yes/no item. From these we derived two measures treated as the study’s focal active-living outcome: a binary indicator of whether the owner had ever taken part in any canine sport, and a breadth count of the number of distinct sports undertaken (range 0–13). Because these variables were not part of the engagement index, they function as external outcomes rather than as a restatement of the measure.

#### 2.2.3 Sociodemographic and structural variables

Place of residence was recorded in four categories (village, town, county seat, capital) and, for the contrast of greatest structural interest, also collapsed to a binary capital-versus-elsewhere indicator. Gender was coded 0 = female, 1 = male, and age was recorded in six ordered bands.

#### 2.2.4 Relative health-related expenditure

A single item asked respondents to compare how much they spent on their own health versus their dog’s health, with four options: more on the dog, more on themselves, the same on both, or neither. The item was shown conditionally and is available for *n* = 533. For analysis it was dichotomised as prioritising the dog’s health versus any other answer.

### 2.3 Analytic strategy

#### 2.3.1 Descriptive analysis

Sample characteristics and the distribution of the engagement items were summarised with frequencies, proportions, means, and standard deviations.

#### 2.3.2 Measurement properties of the engagement index

Before the index was used, its measurement properties were examined. Internal consistency was assessed with Cronbach’s α and McDonald’s ω (from a one-factor maximum-likelihood solution), together with corrected item–total correlations and the full inter-item correlation matrix. Dimensionality was examined through the Kaiser–Meyer–Olkin measure, Bartlett’s test, the eigenvalues of the correlation matrix, and one- and three-factor maximum-likelihood factor solutions, the latter with oblimin rotation to check whether the affective, dispositional, and behavioural facets specified in advance were recoverable and how strongly they correlated. The distribution of the index (skewness, range, quartiles) was inspected to confirm that it could sensibly be treated as a continuous predictor.

#### 2.3.3 Primary analysis: canine sport participation

The primary analysis was a binary logistic regression with lifetime canine sport participation as the outcome. A covariate-only model (Model 0: gender, capital-city residence, age group) was compared with a model adding the engagement index per standard deviation (Model 1), and with a model replacing the index by its three facet subscales (Model 2), each also per standard deviation. Models are reported as odds ratios with 95% confidence intervals, McFadden’s pseudo-R^2^, the area under the ROC curve (AUC), and, for the engagement index, the average marginal effect on the probability of participation. Two checks addressed the functional form of the engagement effect: a quadratic term was added to Model 1, and tertile indicators of the index were added to the linear term, so that a significant improvement would signal departure from a smooth monotone relationship. Calibration was inspected by comparing observed and predicted participation across deciles of predicted probability.

#### 2.3.4 Further outcomes

Breadth of participation (number of distinct sports, 0–13) was strongly right-skewed with a spike at zero, so it was analysed in a hurdle logic: the participation model above accounts for the zero versus non-zero contrast, and a negative-binomial regression among participants only (*n* = 465), with the same predictors, describes how breadth relates to engagement once an owner has taken part in at least one sport. Because 29 respondents reported having taken part in all thirteen sports and had, on average, low engagement scores—a pattern consistent with careless responding—all breadth analyses were repeated with these respondents excluded. Bivariate associations between engagement and breadth used Spearman’s ρ. Place of residence was examined by one-way ANOVA across the four residence categories and by a logistic regression of capital-city residence on engagement, gender, and age. Relative health-related expenditure was examined in a logistic regression of dog-prioritised spending on engagement and the sociodemographic covariates in the subsample with the item (*n* = 533).

The analyses reported in the main text were carried out in Python 3.12 using pandas 3.0, NumPy 2.4, SciPy 1.17, statsmodels 0.15, and factor_analyzer 0.5. The supplementary latent profile analysis, its distal-outcome and sensitivity checks, and the profile-based regression were carried out in R version 4.4.2 using reproducible analysis scripts; the profile solution was reproduced independently in Python for the model comparison reported in the Results. The engagement index used here is the raw 1–5 mean of the ten items; the alternative composite formed by averaging the z-standardised items, used in an earlier version of this analysis, correlates with it at *r* = 0.9996 and yields practically identical estimates (e.g., OR per SD for canine sport participation 2.24 vs. 2.27).

#### 2.3.5 Supplementary latent profile analysis

In an earlier stage of the analysis, a latent profile analysis (LPA) was estimated on the ten standardised items to explore whether owners fell into discrete engagement types (15–17, 27–30). A stable three-profile solution emerged, but the profiles differed only in degree—every item was ordered the same way across them, with no crossover—so they represented positions on a single continuum rather than qualitatively distinct groups. On that evidence we treat engagement as a continuous quantity in the main analysis. The LPA, its distal-outcome tests with the three-step Bolck–Croon–Hagenaars correction (31–35), a categorical latent class sensitivity analysis (36, 37), and a formal comparison of the profile-based and continuous specifications of the participation model are reported in the Supplementary material.

### 2.4 Ethics statement

The study was approved by the Research Ethics Committee of the Faculty of Economics and Business, University of Debrecen (reference: GTK-KB 001/2023). Participation was voluntary and anonymous, and electronic informed consent was obtained before the questionnaire began.

## 3 Results

### 3.1 Sample characteristics

The 954 respondents were predominantly female (71.2%) and concentrated in towns, county seats, and the capital (75.1% combined). The single largest age band was 18–25 years (34.2%). Long-term ownership was common: 60.6% had kept dogs for more than five years, and single-dog households predominated (64.9%). Table 2 gives the full breakdown.

**Table 2:** Sociodemographic characteristics of the sample (*n* = 954).

Variable/category	*n*	%
Gender — Female	679	71.2
Male	275	28.8
Age group — 18–25	326	34.2
26–35	174	18.2
36–45	219	23.0
46–55	182	19.1
56–64	37	3.9
65+	16	1.7
Place of residence — Village	237	24.8
Town	436	45.7
County seat	168	17.6
Capital (Budapest)	113	11.8

The ten items were complete for all respondents. As Table 3 shows, the affective and dispositional items sat highest—preference for animal company (*M* = 3.74), leisure time with the dog (*M* = 3.68), and active-leisure preference (*M* = 3.58)—while the more specialised activity items, such as canine-sport preference (*M* = 2.62) and running with the dog (*M* = 2.19), were both lower and more spread out. Every retained item carried adequate variance.

**Table 3:** Descriptive statistics, corrected item–total correlations, and one-factor loadings of the ten engagement items (*n* = 954; scale 1–5).

Item	M	SD	Corrected item–total r	One-factor loading
Preference for animal company	3.74	1.22	0.62	0.78
Leisure time with dog	3.68	1.25	0.61	0.77
Active-leisure preference	3.58	1.21	0.55	0.58
Openness to novelty	3.20	1.19	0.56	0.58
Hiking with dog	2.82	1.34	0.59	0.64
Daily sport	2.73	1.39	0.46	0.43
Time in nature	2.71	1.23	0.43	0.42
Canine-sport preference	2.62	1.36	0.63	0.67
Trekking with dog	2.59	1.38	0.56	0.60
Running with dog	2.19	1.38	0.31	0.30

Loadings from a one-factor maximum-likelihood solution (all in the same direction). Cronbach’s *α* = 0.837; McDonald’s *ω* = 0.837.

### 3.2 Measurement properties of the engagement index

The ten items were positively and, for the most part, moderately intercorrelated (Table 4). The mean inter-item correlation was 0.34 (range 0.12–0.77); the two affective items (*r* = 0.77) and the two walking-type activity items, hiking and trekking (*r* = 0.76), formed the closest pairs, whereas running with the dog was the most weakly related to the rest. Internal consistency was good (Cronbach’s *α* = 0.84; McDonald’s *ω* = 0.84), and corrected item–total correlations ranged from 0.31 (running) to 0.63 (canine-sport preference). The data were suitable for factor analysis [KMO = 0.82; Bartlett’s test χ^2^(45) = 3758.2, *p* < 0.001]. The first eigenvalue (4.17) accounted for 41.7% of the variance and was more than three times the second (1.34), and all ten items loaded in the same direction on a single factor (0.30–0.78; Table 3). A three-factor oblimin solution recovered the facets specified in advance—affective (leisure time with the dog 0.96, animal company 0.74), behavioural (trekking 0.91, hiking 0.83), and dispositional (daily sport 0.72, time in nature 0.62, openness to novelty 0.60, active-leisure preference 0.54)—with canine-sport preference and running loading diffusely across factors; the three factors were themselves correlated at 0.39 to 0.49, and the corresponding subscales at 0.43 to 0.53. The items therefore measure related facets of one broad engagement dimension. The index was approximately normally distributed (*M* = 2.99, SD = 0.83, range 1.00–5.00, skewness = −0.10; quartile cut-points 2.40, 3.00, 3.60), which supports treating it as a continuous predictor.

**Table 4:** Inter-item correlations of the ten engagement items (*n* = 954).

Item	1	2	3	4	5	6	7	8	9	10
1. Active-leisure preference	—									
2. Leisure time with dog	0.42	—								
3. Preference for animal company	0.44	0.77	—							
4. Canine-sport preference	0.37	0.51	0.47	—						
5. Daily sport	0.42	0.20	0.25	0.38	—					
6. Time in nature	0.34	0.20	0.25	0.31	0.39	—				
7. Openness to novelty	0.48	0.42	0.45	0.35	0.41	0.47	—			
8. Running with dog	0.22	0.15	0.12	0.29	0.26	0.13	0.13	—		
9. Hiking with dog	0.26	0.45	0.44	0.46	0.21	0.23	0.28	0.27	—	
10. Trekking with dog	0.25	0.38	0.39	0.46	0.18	0.23	0.27	0.26	0.76	—

Pearson correlations; all *p* < 0.001. Lower triangle shown.

### 3.3 Engagement and canine sport participation

Just under half of the sample (48.7%) had taken part in at least one canine sport. Owners who had done so scored markedly higher on engagement (*M* = 3.30, SD = 0.79) than those who had not [*M* = 2.69, SD = 0.75; t(952) = 12.14, *p* < 0.001; Cohen’s *d* = 0.79; point-biserial *r* = 0.37]. The association was graded across the whole range of the index (Table 5): lifetime participation rose from 25.8% in the lowest quartile of engagement to 41.0, 56.4, and 77.1% in the second, third, and top quartiles, and the median number of sports undertaken from 0 in the two lower quartiles to 1 and 2 in the two upper ones. Capital-city residence and dog-prioritised health spending rose across the same quartiles.

**Table 5:** Canine sport participation, breadth, capital-city residence, and dog-prioritised health expenditure by quartile of the engagement index.

Engagement quartile (range)	*n*	Canine sport participation (%)	Median no. of sports	Lives in capital (%)	Prioritises dog’s health (%)ᵃ
Q1 (1.00–2.40)	260	25.8	0	6.5	15.3
Q2 (2.41–3.00)	249	41.0	0	8.0	18.5
Q3 (3.01–3.60)	227	56.4	1	15.0	29.3
Q4 (3.61–5.00)	218	77.1	2	19.3	31.7

ᵃSubsample with the expenditure item (*n* = 533).

Table 6 reports the logistic regression models. With sociodemographic covariates alone (Model 0), capital-city residence was the only significant predictor of participation [OR = 1.89, 95% CI (1.25, 2.85)], and the model explained little (McFadden’s pseudo-R^2^ = 0.011; AUC = 0.563). Adding the engagement index (Model 1) improved fit substantially [likelihood-ratio χ^2^(1) = 128.6, *p* < 0.001; pseudo-R^2^ = 0.108; AUC = 0.723]. Each standard deviation of engagement was associated with 2.27 times the odds of lifetime participation [95% CI (1.94, 2.64)], or 2.69 times the odds per scale point on the 1–5 metric; the average marginal effect was 17.6 percentage points per standard deviation [95% CI (15.0, 20.1)]. The unadjusted estimate was almost identical (OR = 2.29 per SD), so the association was not an artefact of the covariates. For a female owner outside the capital in the median age band, the predicted probability of participation was 26% one standard deviation below the mean of engagement, 45% at the mean, 65% one standard deviation above it, and 81% two standard deviations above. Once engagement was in the model, capital-city residence no longer predicted participation [OR = 1.36, 95% CI (0.87, 2.10)], women had somewhat lower odds than men [OR = 0.75, 95% CI (0.55, 1.01), *p* = 0.058], and age was unrelated. Calibration was adequate: across deciles of predicted probability, observed participation ran from 26.5 to 82.3% against predicted values of 17.8 to 79.5%, with no systematic over- or under-prediction in the upper half of the distribution.

**Table 6:** Binary logistic regression models predicting lifetime canine sport participation (*n* = 954).

Predictor	Model 0: OR (95% CI)	Model 1: OR (95% CI)	Model 2: OR (95% CI)
Engagement index (per SD)	—	2.27 (1.94–2.64)***	—
Affective facet (per SD)	—	—	1.31 (1.09–1.57)**
Dispositional facet (per SD)	—	—	1.29 (1.08–1.53)**
Behavioural facet (per SD)	—	—	1.69 (1.44–1.99)***
Female (vs. male)	0.80 (0.60–1.06)	0.75 (0.55–1.01)	0.70 (0.51–0.97)*
Capital (vs. elsewhere)	1.89 (1.25–2.85)**	1.36 (0.87–2.10)	1.27 (0.81–1.99)
Age group	1.06 (0.96–1.17)	1.07 (0.96–1.18)	1.05 (0.95–1.17)
McFadden’s pseudo-R^2^	0.011	0.108	0.115
AUC	0.563	0.723	0.729
AIC	1316.0	1189.4	1184.2

OR, odds ratio; CI, confidence interval; AUC, area under the ROC curve. Facets in Model 2 are subscale means entered per standard deviation (affective: leisure time with dog, animal company; dispositional: active-leisure preference, canine-sport preference, daily sport, time in nature, openness to novelty; behavioural: running, hiking, trekking with dog). *p < 0.05; **p < 0.01; ***p < 0.001.

The relationship was monotone but not exactly linear on the logit scale. A quadratic term improved fit modestly (*p* = 0.011) and was positive, indicating a slightly convex curve—flat at low engagement, steepest through the middle of the scale—and tertile indicators added to the linear term also improved fit [χ^2^(2) = 10.2, *p* = 0.006], although the BIC favoured the linear specification. Figure 1 shows the shape: observed participation was between 23 and 34% for index values up to 2.5, rose steeply between 2.5 and 4.0, and levelled off at 75–78% above 3.5. The engagement effect is thus concentrated in the middle of the distribution and saturates at the top, but it is present across the full range rather than confined to a single threshold.

**Figure 1:**
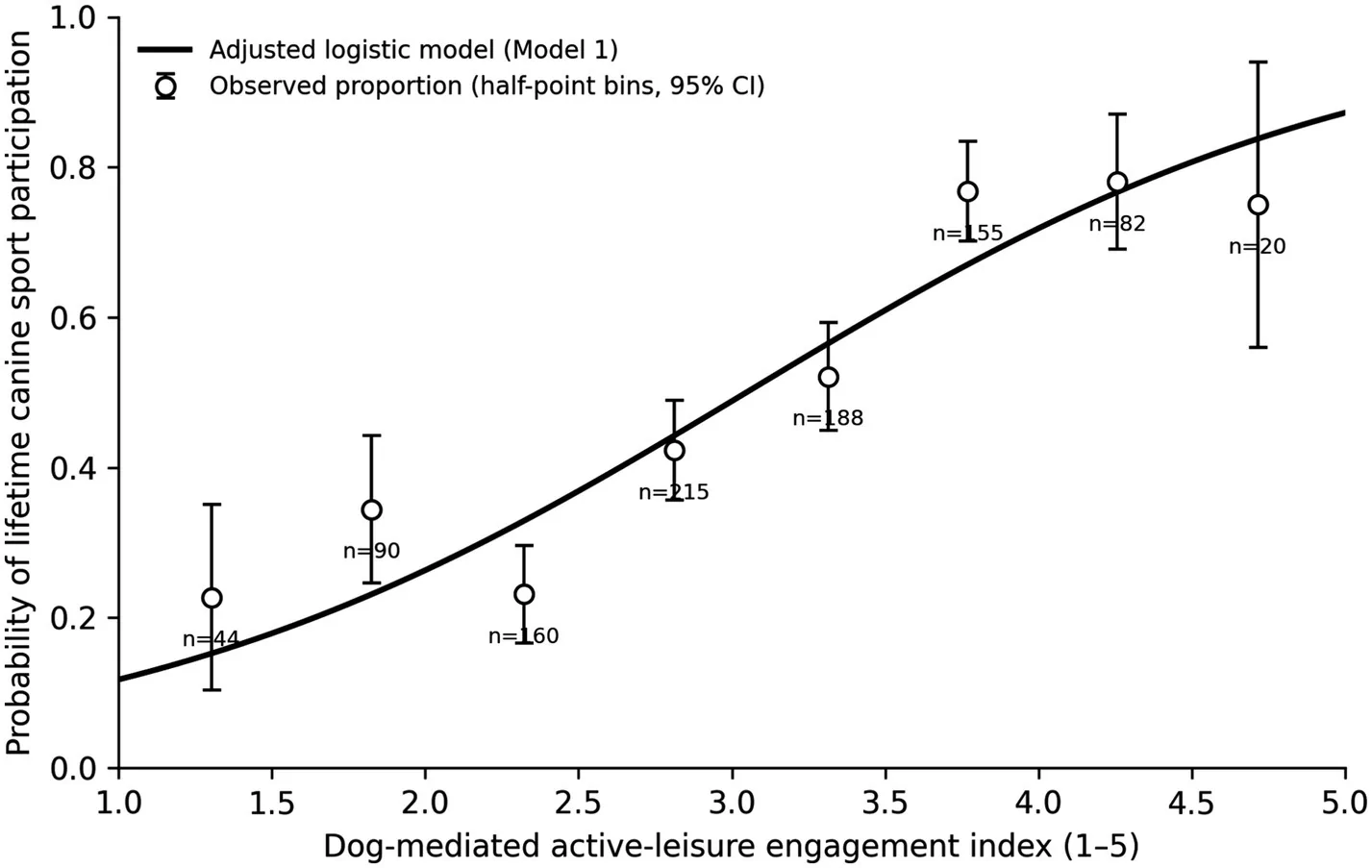
Probability of lifetime canine sport participation across the dog-mediated active-leisure engagement index (1–5). The curve is the average predicted probability from the adjusted logistic model (Model 1) over the observed distribution of gender, residence, and age; circles are observed proportions in half-point bins of the index with 95% confidence intervals and bin sizes.

### 3.4 Which facets of engagement carry the association

When the index was replaced by its three facet subscales (Model 2), all three were independently associated with participation. The behavioural facet—how often the owner ran, hiked, or trekked with the dog—carried the largest coefficient [OR = 1.69 per SD, 95% CI (1.44, 1.99)], with the affective [OR = 1.31, 95% CI (1.09, 1.57)] and dispositional [OR = 1.29, 95% CI (1.08, 1.53)] facets contributing about equally beyond it. The facet model fitted only marginally better than the single-index model (pseudo-R^2^ = 0.115 vs. 0.108; AUC = 0.729 vs. 0.723), so the summary index loses little information, but the pattern indicates that everyday dog-mediated physical activity is the facet most closely tied to organised participation, with the bond and the general active-leisure orientation adding independent increments.

Breadth of participation followed the participation contrast rather than adding to it. Across the whole sample, engagement correlated with the number of sports undertaken (Spearman’s ρ = 0.34, *p* < 0.001), but this association was almost entirely a product of the zero versus non-zero difference: among the 465 participants, engagement and the number of sports were unrelated (ρ = 0.01, *p* = 0.86), and the negative-binomial estimate was, if anything, negative [incidence rate ratio (IRR) = 0.87 per SD, 95% CI (0.82, 0.94)]. This estimate was driven by the 29 respondents who reported all thirteen sports and had low engagement scores (*M* = 2.25 vs. 3.30 among other participants). With those respondents excluded, breadth correlated weakly and positively with engagement among the remaining participants [*ρ* = 0.15, *p* = 0.001; IR*R* = 1.07 per SD, 95% CI (1.00, 1.16), *p* = 0.067], and the participation odds ratio was essentially unchanged (OR = 2.70 per SD). Engagement therefore distinguishes owners who take up organised canine sport from those who do not far more sharply than it distinguishes, among participants, those who try many sports from those who try few.

### 3.5 Structural and sociodemographic correlates of engagement

Engagement rose across the residence gradient: mean index scores were 2.85 (SD = 0.83) in villages, 2.94 (0.83) in towns, 3.04 (0.79) in county seats, and 3.35 (0.73) in the capital (one-way ANOVA *F*(3, 950) = 10.37, *p* < 0.001; Kruskal–Wallis H(3) = 30.6, *p* < 0.001). The contrast of greatest structural interest, capital versus elsewhere, amounted to 0.41 scale points [t(952) = 5.08, *p* < 0.001], or about half a standard deviation. In a logistic regression of capital-city residence on engagement, gender, and age, each standard deviation of engagement was associated with 1.69 times the odds of living in the capital [95% CI (1.36, 2.09), *p* < 0.001], and an ordinal model across the four residence categories gave a proportional odds ratio of 1.33 per SD (*p* < 0.001). Engagement, in other words, is not spread evenly across the country but concentrates in the capital. Gender and age were not associated with engagement [women *M* = 3.00 vs. men *M* = 2.95, t(952) = 0.94, *p* = 0.35; Spearman’s ρ with age ban*d* = 0.04, *p* = 0.27], so neither confounds the associations reported above. Read together with Model 1, in which capital-city residence stopped predicting participation once engagement was included, the pattern suggests that the higher participation of metropolitan owners operates largely through their higher engagement rather than alongside it.

### 3.6 Relative health-related expenditure

Among the subsample shown the expenditure item (*n* = 533), 21.8% reported spending more on their dog’s health than on any other option. These owners scored higher on engagement (*M* = 3.06, SD = 0.82) than the rest [*M* = 2.74, SD = 0.82; t(531) = 3.68, *p* < 0.001], and the proportion prioritising the dog rose from 15.3% in the lowest engagement quartile to 31.7% in the highest (Table 5). In a logistic regression with the sociodemographic covariates, each standard deviation of engagement was associated with 1.40 times the odds of prioritising the dog’s health [95% CI (1.13, 1.74), *p* = 0.002; unadjusted OR = 1.47]; capital-city residence was also independently associated with dog-prioritised spending [OR = 3.13, 95% CI (1.41, 6.95), *p* = 0.005]. The model explained little variance (pseudo-R^2^ = 0.038), so this is corroborating rather than load-bearing evidence, but its direction is consistent with the behavioural and structural findings.

### 3.7 Comparison with a profile-based specification

For completeness, the supplementary latent profile analysis is compared here with the continuous specification. The three profiles (Engaged, 40.3%; Moderate, 39.8%; Disengaged, 19.9%) were almost entirely a coarsening of the index: the index explained 77% of the variance in profile membership (η^2^ = 0.77), the profile mean index scores were 3.78, 2.75, and 1.87, and adjacent profiles overlapped in index range (Supplementary Table S5). As predictors of participation, the two specifications performed equivalently (profile model: pseudo-R^2^ = 0.109, AIC = 1190.4; index model: 0.108, 1189.4). Each added something to the other in a likelihood-ratio test—the index over the profiles [χ^2^(1) = 17.7, *p* < 0.001] because it retained within-profile variation that still predicted participation (Spearman’s ρ = 0.17 within both the Engaged and the Moderate profile), and the profiles over the linear index [χ^2^(2) = 18.7, *p* < 0.001] because they approximate the convexity noted above. A cubic polynomial in the index captured that shape with equal parsimony (AIC = 1180.8). Nothing in the profile solution, therefore, is recoverable only by treating owners as members of discrete groups; the continuous index reproduces the substantive findings and preserves information the profiles discard.

## 4 Discussion

This study asked how strongly dog owners’ active-leisure engagement is associated with organised physical activity, and whether that engagement is patterned by place and by household spending priorities. Measured with a ten-item index that proved internally consistent and essentially unidimensional, engagement was strongly and continuously associated with lifetime participation in canine sport: each standard deviation more than doubled the odds of participation, the predicted probability rose from about a quarter to about two-thirds across a two-standard-deviation span, and the association held unchanged when gender, residence, and age were controlled. Because participation was measured separately from the engagement items, the contrast is genuine external evidence that the index captures something behaviourally real rather than an artefact of its own content.

### 4.1 Engagement as a continuous dimension

The most important interpretive point concerns the form of the relationship. A supplementary latent profile analysis recovered three stable groups, but they differed only in degree, with every item ordered the same way across them and no crossover, and the continuous index reproduced everything the profiles showed while retaining variation within them that still predicted participation. Dog owners do not, on this evidence, fall into qualitatively different kinds of engagement in the way that person-centred typologies sometimes reveal (18); they occupy positions along a single dimension on which the affective, dispositional, and behavioural facets rise together. The affective items had the highest loadings, which suggests that the bond with the animal lies closer to the core of that dimension than any particular activity does (38–40), while the facet-level model showed that everyday dog-mediated physical activity is the component most closely tied to organised participation. Two consequences follow. First, differences between owners are differences of degree, so any cut-point used to describe them—quartiles here, profiles in the Supplement—is a descriptive convenience rather than a discovery. Second, the relationship is not uniform across the scale: it is flat at the low end, steepest through the middle, and saturates at the top, where three-quarters of owners have already taken part in organised sport. The room for movement lies in the middle of the distribution.

### 4.2 Engagement and organised physical activity

The clearest behavioural correlate of engagement was participation in canine sport, the study’s focal active-living outcome. Lifetime participation tripled between the lowest and highest quartiles of engagement, and the adjusted odds ratio per standard deviation was above two. Breadth of participation told a different story. Across the sample it rose with engagement, but among owners who had taken part in at least one sport the number of sports was essentially unrelated to engagement once a small group of respondents who ticked every sport—and whose low engagement scores mark them as probable careless responders—was set aside. We therefore rest the conclusion on the participation contrast, which was robust to that exclusion and to the choice of functional form, and treat breadth as a secondary indicator that adds little beyond it.

Substantively, this fits the picture emerging from the broader literature on dogs and physical activity, in which ownership creates an opportunity for movement that only some owners convert into sustained, organised activity (41, 42). It is useful to think of canine sport as the demanding end of that conversion. Ordinary dog walking, on which most of the dog-and-health literature has concentrated, is a low-threshold behaviour available to nearly every owner; organised canine sport is not (2, 6, 43). It asks for time, travel, training, club membership, and a degree of identification with the activity that a high level of engagement supplies and a low one does not. The shape of the curve is, on this reading, exactly what one would expect: moving into organised sport is a large step rather than a small one, taken disproportionately by owners for whom the dog is already central to leisure (6, 43, 44), and once engagement is high enough the step has usually been taken. The implication is that the relationship between dogs and physical activity is unlikely to be uniform across the owning population; it is structured by where an owner already sits on the engagement dimension.

One covariate deserves comment in its own right. Although the sample was predominantly female, men had somewhat higher odds than women of having taken part in canine sport once engagement was held constant, and the difference reached significance in the facet-level model. The associative design does not let us adjudicate why, but the result is consistent with documented gender patterning in competitive and organised leisure participation more generally (45, 46), and it cautions against assuming that a strong owner–dog bond, which did not differ by gender in these data, maps directly onto organised sporting activity. Bond and organised participation are related but distinct, and they need not be patterned the same way across subgroups.

### 4.3 The structural patterning of engagement

Engagement was not spread evenly across the country. Owners in the capital scored about half a standard deviation higher on engagement than owners elsewhere, and engagement rose monotonically from villages through towns and county seats to the capital. Two readings are compatible with the data. Access is the more obvious one: the clubs, training grounds, instructors, and events that organised canine sport depends on are concentrated in larger urban centres, so a metropolitan owner faces lower practical barriers to engagement than a rural one (47). Selection is the other: people already oriented toward active, organised leisure may gravitate to, or remain in, cities for reasons unconnected to their dogs, so that the urban concentration reflects who lives where rather than what the city affords. The adjusted model offers a modest clue between the two. Capital-city residence predicted canine sport participation on its own, but not once engagement entered the model, which is consistent with residence operating largely through engagement rather than as an independent driver of activity. We advance this as a hypothesis the design can support, not as a settled mechanism; a single cross-sectional association cannot separate access from selection, and disentangling them would require either longitudinal data or a design that varies access while holding disposition fixed.

### 4.4 The economic signature of engagement

A further line of external evidence came from how owners weighed spending on their dog’s health against spending on their own. The tendency to prioritise the dog rose with engagement, roughly doubling between the lowest and highest quartiles, and remained associated with engagement after adjustment for residence, gender, and age. This was the weakest of the three external associations, and we treat it accordingly—as corroboration rather than as a load-bearing finding. Its direction is nonetheless coherent. Owners for whom the dog is central enough to structure their leisure also appear more willing to place the animal’s health ahead of their own in household spending, which extends the behavioural portrait of engagement into the domain of resource allocation (48). That a purely economic indicator, measured separately and on only part of the sample, lines up with the behavioural and structural evidence adds a little to the credibility of engagement as a real organising dimension rather than a statistical convenience.

### 4.5 Overall interpretation and implications

Taken together, the findings describe engagement as the realised use of an opportunity. A dog offers nearly every owner a route into physical activity, but whether that route is taken depends on a bundle of dispositional, affective, and behavioural conditions that co-vary, and that the index summarises in a single score. Owners at the high end are already disproportionately metropolitan, already bonded to their animals, and already participating in organised sport; the leverage for promoting active living through dogs lies not with them but with the large middle and lower range of the distribution—the three-fifths of the sample below the third quartile cut-point—where organised canine activity is still the exception.

This reframing has practical purchase. Efforts to use dogs as a vehicle for population physical activity often proceed as though the binding constraint were ownership itself; our results suggest that, among owners, the binding constraints are engagement and access. The most promising target is arguably the middle of the engagement distribution: owners who already keep and walk a dog, and who sit only modestly below the level at which participation becomes the norm, but who have not crossed into organised activity. The steepness of the curve in exactly that region implies that a modest shift in engagement there corresponds to a large change in the probability of participation. Lowering the threshold for such owners—through more widely distributed clubs, introductory or low-commitment formats, taster events, and outreach to owners who walk but do not yet take part in structured activity—would address the part of the distribution where the room for movement is greatest (49). The geographic finding sharpens the point: because organised provision and engagement both concentrate in the capital, programmes delivered only where participation is already high risk widening the gap in active living between owner subgroups rather than closing it. A public-health framing of dogs as a route to activity therefore needs to attend not only to whether people own dogs but to where the supporting infrastructure sits and to how engaged owners already are.

## 5 Limitations

Several limitations bound these conclusions. First, and most importantly, the design is cross-sectional, so every association reported here is just that—an association. We cannot tell whether an active-leisure disposition leads owners into canine sport, whether participation cultivates the disposition, or whether some third characteristic underlies both. The directional language of cause has no place in the interpretation, and we have worked to keep it out throughout.

Second, all measures are self-reported, including the lifetime record of canine sport participation, which is open to recall error and to social-desirability pressure in a sample of engaged owners. The breadth measure is the more vulnerable of the two: a small group of respondents reported every one of the thirteen sports while scoring low on engagement, a pattern that points to careless or acquiescent responding, and the association between engagement and breadth among participants changed sign when they were excluded. We have therefore based the substantive conclusions on the participation contrast, which was unaffected by their exclusion, and report the breadth results as secondary.

Third, recruitment used non-probability quota sampling, and the realised sample was predominantly female and entirely Hungarian. Both the measurement structure of the index and the external associations may not transfer cleanly to other owner populations or national contexts, where the canine sport sector, its geography, and the gendering of participation may differ. Replication elsewhere is needed before the association is treated as general.

Fourth, the engagement index is an unweighted mean of ten items that were selected on theoretical and distributional grounds rather than drawn from a validated instrument. Its internal consistency was good and its structure essentially unidimensional, and the facet-level model showed that the summary loses little predictive information, but the index has not been validated against established measures of the human–animal bond or of leisure orientation, and its item content weights everyday walking-type activity more heavily than the affective bond. Different item sets could shift the strength, though not plausibly the direction, of the associations reported here.

Fifth, the relationship between engagement and participation departed modestly from linearity on the logit scale, being steepest in the middle of the index and flattening at the top. We report this shape descriptively and rest the main estimates on the linear specification, which the BIC favoured; readers interested in the location of the steepest region should treat the half-point bins in Figure 1 as descriptive rather than as evidence of a threshold.

Sixth, the relative-expenditure item was available only for a subsample and is a single self-report measure.

## 6 Conclusion and future directions

A cross-sectional analysis of Hungarian dog owners showed that dog-mediated active-leisure engagement, measured as a single internally consistent index, is strongly and continuously associated with organised physical activity. Owners high in engagement were set apart above all by their participation in canine sport, with the structural and economic correlates of engagement pointing in the same direction but carrying less weight. The association was graded across the full range of the scale, steepest in its middle, and saturated at the top, which locates the potential for promoting active living through dogs in the large, currently under-engaged majority rather than in the already-active minority. Read this way, the study reframes a familiar question. The issue is less whether owning a dog is associated with physical activity—on average, modestly, it is—than which owners convert ownership into sustained, organised activity, and under what conditions.

Three directions follow. Longitudinal designs are needed to establish whether engagement and organised activity reinforce one another over time, and in which direction the influence mainly runs. Intervention work could test whether lowering the practical and geographic barriers to organised canine activity actually raises engagement and participation, particularly outside metropolitan centres, where both provision and participation are currently thin. And replication in other national contexts, ideally with a validated engagement measure, would show how far the continuous association reported here, and its concentration of physical activity at the engaged end, generalises beyond the single setting studied. Pursuing these questions would help turn a descriptive association into a basis for using companion dogs, deliberately and equitably, as a route to a more active life.

## Data Availability

The raw data supporting the conclusions of this article will be made available by the authors, without undue reservation.
