# Supplementary material for: Dog-mediated active-leisure engagement and organised canine sport: a cross-sectional analysis of Hungarian dog owners
Source: Front Vet Sci. 2026 Sep 25;13:1912638. doi: 10.3389/fvets.2026.1912638 (PMC13650940; doi:10.3389/fvets.2026.1912638)
Supplement: Supplementary file 1 [file Data_Sheet_1.docx]

**Supplementary Material**

*Dog-Mediated Active-Leisure Engagement and Organised Canine Sport: A Cross-Sectional Analysis of Hungarian Dog Owners*

This supplement reports the latent profile analysis (LPA) that preceded the continuous analysis presented in the main text, its distal-outcome and sensitivity checks, and a formal comparison between the profile-based and the continuous specification of the model predicting canine sport participation. The profile-based analyses were carried out in R 4.4.2; the profile solution was reproduced independently in Python 3.12 (identical class sizes, entropy, and distal proportions) for the model comparison in Section S4.

# S1. Latent profile analysis: estimation and model selection

The ten engagement items were standardised to z-scores before estimation. The model was a Gaussian finite mixture with class-invariant diagonal covariances—within-class variances held equal across classes and within-class covariances fixed at zero (the EEI parameterisation). This specification was adopted after a model allowing class-varying diagonal covariances proved numerically unstable, failing to converge cleanly once three or more profiles were requested. The five-point items were treated as continuous; the influence of this simplification is tested in Section S3.

Models with two to six profiles were estimated, each from 150 random starts, with the maximum-likelihood solution retained at every level. Selection drew on the Bayesian information criterion (BIC), relative entropy, the size of the smallest class, substantive interpretability, and—decisively—the stability of the solution across random starts, indexed by the proportion of starts reaching the maximum-likelihood solution and by the mean adjusted Rand index (ARI) of each start’s modal classification against that solution. Respondents were assigned to the profile with the highest posterior probability.

As often happens with large samples, the BIC kept improving as profiles were added, with no clear elbow (Table S1; Figure S1a). Stability across random starts was the deciding criterion. Every one of the 150 starts for the three-profile model converged on the same solution (100%; mean ARI = 1.00), whereas only about half of the starts for the four-profile model reached the best solution (52%; mean ARI = 0.75), the remainder settling on materially different configurations (Figure S1b)—a pattern that points to a multimodal likelihood surface and a poorly identified fourth profile. The three-profile solution was therefore retained. It showed satisfactory classification certainty (entropy = .846), with average posterior probabilities of .95, .92, and .93 for the three profiles and no class smaller than one-fifth of the sample.

**Table S1. Latent profile model fit and stability (maximum-likelihood solution per level; n = 954).**

| **Profiles (K)** | **BIC** | **Entropy** | **Smallest class (%)** | **Starts at ML (%)** | **Mean ARI** |
| --- | --- | --- | --- | --- | --- |
| 2 | −25199.8 | .847 | 48.8 | 100 | 1.00 |
| 3 (selected) | −24586.0 | .846 | 19.9 | 100 | 1.00 |
| 4 | −24307.6 | .849 | 15.0 | 52 | 0.75 |
| 5 | −24159.2 | .822 | 14.5 | 55 | 0.80 |
| 6 | −23999.6 | .848 | 13.0 | 20 | 0.65 |

Note. BIC reported on the +2logL − k·log(n) scale (higher = better). EEI parameterisation; items standardised; 150 random starts per level. “Starts at ML” = proportion of random starts reaching the maximum-likelihood solution; “Mean ARI” = mean adjusted Rand index of each start’s modal classification against that solution.

Figure S1. Model selection across two- to six-profile solutions (EEI parameterisation; 150 random starts per level). (a) BIC on the +2logL − k·log n scale (higher = better), which improves monotonically with no clear elbow. (b) Solution stability: the percentage of random starts reaching the maximum-likelihood solution, with the mean adjusted Rand index annotated. The three-profile solution was reached by every start; the four-profile solution by about half.

# S2. Characteristics of the profiles

The standardised item means (Table S2; Figure S2) describe a graded structure rather than three qualitatively separate types: the profiles differ in degree, with every item ordered the same way across them and no crossover in which one group is high on what another is low on. The labels below are descriptive conveniences.

The Engaged profile (n = 384, 40.3%) scored above the sample mean on all ten items. The separation was widest on the activity and preference items—canine-sport preference and trekking with the dog both sat around 0.8 standard deviations above the mean, with hiking close behind—and was reinforced by the affective items, leisure time with the dog (0.69) and preference for the company of animals (0.67). The Moderate profile (n = 380, 39.8%) sat close to the mean throughout: essentially average on the affective items (0.01 and 0.04) and modestly below the mean on the more demanding behavioural and dispositional items, such as canine-sport preference (−0.38), trekking (−0.45), and hiking (−0.38). The Disengaged profile (n = 190, 19.9%) fell well below the mean on every item, and most steeply on the affective ones: preference for the company of animals (−1.41) and leisure time with the dog (−1.39) both lay roughly 1.4 standard deviations below average, followed by active-leisure preference (−0.92) and openness to novelty (−0.90).

**Table S2. Standardised item means by profile.**

| **Item** | **Engaged (n = 384)** | **Moderate (n = 380)** | **Disengaged (n = 190)** |
| --- | --- | --- | --- |
| Active-leisure preference | 0.54 | −0.08 | −0.92 |
| Leisure time with dog | 0.69 | 0.01 | −1.39 |
| Preference for animal company | 0.67 | 0.04 | −1.41 |
| Canine-sport preference | 0.82 | −0.38 | −0.85 |
| Daily sport | 0.42 | −0.14 | −0.56 |
| Time in nature | 0.39 | −0.07 | −0.63 |
| Openness to novelty | 0.54 | −0.08 | −0.90 |
| Running with dog | 0.36 | −0.17 | −0.38 |
| Hiking with dog | 0.80 | −0.38 | −0.84 |
| Trekking with dog | 0.82 | −0.45 | −0.72 |

Note. Class means on standardised (z-score) items; maximum-likelihood EEI solution; entropy = .846.

Figure S2. Standardised (z-score) means of the ten active-leisure engagement items for the three profiles. The dashed line marks the sample mean. Every item is ordered the same way across profiles, with the steepest separation of the Disengaged group on the affective items.

# S3. Profile differences on the external variables

Between-profile differences were examined in three layers. First, using modal class assignment, with chi-square tests and Cramér’s V for categorical outcomes and Kruskal–Wallis tests for ordinal outcomes. Second, to guard against bias from treating modal assignments as error-free, the associations were re-estimated with the three-step Bolck–Croon–Hagenaars (BCH) correction, which weights each case by the inverse of the estimated classification-error matrix, with inference from 2,000 nonparametric bootstrap replications. Third, canine sport participation was modelled in an adjusted binary logistic regression with profile membership and the sociodemographic variables as predictors.

Lifetime participation in canine sport ran at 71.4% among Engaged owners, against 35.0% in the Moderate and 30.5% in the Disengaged group (χ²(2) = 132.5, p < .001, Cramér’s V = .373). The median number of distinct sports was 2 in the Engaged group and 0 in both other groups (Kruskal–Wallis H = 115.3, p < .001). Under the BCH adjustment the participation estimates were .73, .33, and .30, and the Engaged-versus-Disengaged contrast remained firmly positive (95% bootstrap CI for the difference [.35, .52]). Place of residence tracked profile membership (χ²(6) = 48.1, p < .001, V = .159): 19.8% of Engaged owners lived in the capital, against 8.4% of Moderate and 2.6% of Disengaged owners (BCH Engaged-versus-Disengaged 95% CI [.13, .23]). Gender (χ²(2) = 7.6, p = .022, V = .089) and age (Kruskal–Wallis H = 8.1, p = .018) differed only marginally. Among the subsample with the expenditure item (n = 533; 158, 230, and 145 across profiles), the share prioritising the dog’s health was 32.9%, 17.8%, and 15.9% (χ²(2) = 16.6, p < .001, V = .176; BCH estimates .34, .17, .16; Engaged-versus-Disengaged 95% CI [.08, .28]).

**Table S3. Profile differences on the external variables (modal assignment and BCH-corrected estimates).**

| **Outcome** | **Engaged** | **Moderate** | **Disengaged** | **Test (modal)** | **BCH estimates** | **BCH E–D 95% CI** |
| --- | --- | --- | --- | --- | --- | --- |
| Canine sport participation (%) | 71.4 | 35.0 | 30.5 | χ²(2) = 132.5, p < .001; V = .373 | .73 / .33 / .30 | [.35, .52] |
| Median no. of sports | 2 | 0 | 0 | H = 115.3, p < .001 | — | — |
| Lives in capital (%) | 19.8 | 8.4 | 2.6 | χ²(6) = 48.1, p < .001; V = .159 | — | [.13, .23] |
| Prioritises dog’s health (%) | 32.9 | 17.8 | 15.9 | χ²(2) = 16.6, p < .001; V = .176 | .34 / .17 / .16 | [.08, .28] |

Note. E–D = Engaged versus Disengaged. Residence test uses the four-category variable; percentages refer to capital-city residence. Expenditure results are for the subsample with the item (n = 533).

Categorical sensitivity analysis. To check whether modelling the Likert items as continuous had shaped the findings, the typology was re-estimated with a categorical latent class analysis treating each item as an unordered five-category variable. The categorical model recovered a stable three-class structure (every random start reaching the same solution) and agreed moderately with the continuous solution at the level of individual assignment (ARI = .51). The external associations held at near-identical effect sizes under the categorical assignment: canine sport participation (V = .374), capital-city residence (V = .202), and dog-prioritised expenditure (V = .189), all with the same ordering across profiles.

**Table S4. Binary logistic regression predicting lifetime canine sport participation from profile membership (n = 954).**

| **Predictor** | **OR** | **95% CI** | **p** |
| --- | --- | --- | --- |
| Engaged (vs. Disengaged) | 5.72 | 3.87–8.46 | < .001 |
| Moderate (vs. Disengaged) | 1.23 | 0.84–1.79 | .283 |
| Female (vs. male) | 0.68 | 0.50–0.92 | .012 |
| Capital (vs. elsewhere) | 1.22 | 0.78–1.91 | .374 |
| Age group | 1.04 | 0.94–1.16 | .432 |

Note. Reference category for profile = Disengaged. McFadden’s pseudo-R² = .109; AIC = 1190.4.

# S4. Comparison of the profile-based and continuous specifications

Table S5 relates the three profiles to the continuous engagement index used in the main text (the raw 1–5 mean of the ten items). The index explained 77% of the variance in profile membership (one-way ANOVA F(2, 951) = 1599.6, η² = .77), profile means were widely separated, and adjacent profiles overlapped in index range, as expected when a continuum is cut into ordered segments. Within the Engaged and the Moderate profile, the index remained positively associated with canine sport participation (Spearman’s ρ = .17, p < .001, and ρ = .17, p = .001, respectively; Disengaged: ρ = .05, p = .47), so profile membership discards predictive variation that the index retains.

**Table S5. Profiles in relation to the continuous engagement index.**

| **Profile** | **n (%)** | **Index M** | **Index range** | **Canine sport participation (%)** | **Within-profile ρ (index × participation)** |
| --- | --- | --- | --- | --- | --- |
| Engaged | 384 (40.3) | 3.78 | 2.70–5.00 | 71.4 | .17 (p < .001) |
| Moderate | 380 (39.8) | 2.75 | 1.90–3.60 | 35.0 | .17 (p = .001) |
| Disengaged | 190 (19.9) | 1.87 | 1.00–3.00 | 30.5 | .05 (p = .47) |

Table S6 compares alternative specifications of the engagement effect in the adjusted logistic model of canine sport participation (all models include gender, capital-city residence, and age group). The profile model and the linear index model performed equivalently. Each added something to the other in a likelihood-ratio test: the index over the profiles (χ²(1) = 17.7, p < .001), and the profiles over the linear index (χ²(2) = 18.7, p < .001), the latter because the profile boundaries approximate the mild convexity of the relationship. A cubic polynomial in the index captured that shape with equal parsimony and a lower AIC than the profile model, and adding the profiles to the quadratic model contributed less (χ²(2) = 12.4, p = .002) than adding the quadratic to the profile model (χ²(2) = 17.9, p < .001). The BIC, which penalises parameters more heavily, favoured the linear index over every alternative. Nothing in the profile solution is therefore recoverable only by treating owners as members of discrete groups.

**Table S6. Alternative specifications of the engagement effect in the adjusted logistic model of canine sport participation (n = 954).**

| **Specification of engagement** | **Parameters for engagement** | **Log-likelihood** | **AIC** | **BIC** | **McFadden’s pseudo-R²** |
| --- | --- | --- | --- | --- | --- |
| Linear index (per SD) | 1 | −589.7 | 1189.4 | 1213.7 | .108 |
| Linear + quadratic | 2 | −586.4 | 1184.8 | 1214.0 | .113 |
| Linear + quadratic + cubic | 3 | −583.4 | 1180.8 | 1214.8 | .117 |
| Tertile indicators | 2 | −587.5 | 1187.0 | 1216.2 | .111 |
| Quartile indicators | 3 | −586.6 | 1187.2 | 1221.3 | .112 |
| Three LPA profiles | 2 | −589.2 | 1190.4 | 1219.5 | .109 |
| Linear index + three profiles | 3 | −580.3 | 1174.7 | 1208.7 | .122 |
| Linear + quadratic + three profiles | 4 | −580.2 | 1176.5 | 1215.4 | .122 |

Note. All models include female, capital-city residence, and age group. AIC = Akaike information criterion; BIC = Bayesian information criterion.
